# Diagnosis of Cardiac Involvement in Amyloid A Amyloidosis by Cardiovascular Magnetic Resonance Imaging

**DOI:** 10.3389/fcvm.2021.757642

**Published:** 2021-09-27

**Authors:** Bishwas Chamling, Stefanos Drakos, Michael Bietenbeck, Karin Klingel, Claudia Meier, Ali Yilmaz

**Affiliations:** ^1^Division of Cardiovascular Imaging, Department of Cardiology I, University Hospital Münster, Albert Schweitzer Campus 1, Münster, Germany; ^2^Institute for Pathology and Neuropathology, University Hospital Tübingen, Tübingen, Germany

**Keywords:** AA amyloidosis, cardiac involvement, serum amyloid A (SAA), ECV, cardiovascular magnetic resonance (CMR), mapping—magnetic resonance imaging

## Abstract

**Background:** Diagnosis of cardiac involvement in amyloid A (AA) amyloidosis is challenging since AA amyloidosis is a rare disease and cardiac involvement even less frequent. The diagnostic yield of currently available non-invasive imaging methods is not well-studied and rather limited, and invasive endomyocardial biopsy (EMB) is rarely performed due to the potential risk of this procedure. Cardiovascular magnetic resonance (CMR)-based myocardial tissue characterization by late-gadolinium-enhancement (LGE) imaging and novel-mapping approaches may increase the diagnostic yield in AA amyloidosis.

**Methods:** Two patients with AA amyloidosis in whom cardiac involvement was suspected based on CMR findings and subsequently proven by biopsy work-up are presented. CMR studies were performed on a 1.5-T system and comprised a cine steady-state free precession pulse sequence for ventricular function and a late-gadolinium-enhancement (LGE) sequence for detection of myocardial pathology. Moreover, a modified Look-Locker inversion recovery (MOLLI) T1-mapping sequence was applied in basal, mid and apical short-axes prior to contrast agent administration and ~20 min thereafter to determine native T1 and ECV values.

**Results:** Both patients showed slightly dilated left ventricles (LV) with mild to moderate LV hypertrophy and preserved systolic function. Only a very subtle pattern of LGE was observed in both patients with AA amyloidosis. However, markedly elevated native T1 (max. 1,108 and 1,112 ms, respectively) and extracellular volume fraction (ECV) values (max. 39 and 48%, respectively) were measured in the myocardium suggesting the presence of cardiac involvement - with subsequent EMB-based proof of AA amyloidosis.

**Conclusion:** We recommend a multi-parametric CMR approach in patients with AA amyloidosis comprising both LGE-based contrast-imaging and T1-mapping-based ECV measurement of the myocardium for non-invasive work-up of suspected cardiac involvement. The respective CMR findings may be used as gatekeeper for additional invasive procedures (such as EMB) and as a non-invasive monitoring tool regarding assessment and modification of ongoing treatments.

## Introduction

Amyloidosis is a family of multifaceted, heterogenous diseases based on abnormally folded proteins characterized by pathological accumulation of insoluble, polymeric protein fibrils in the extracellular space of various tissues and organs—sometimes leading to organ dysfunction, organ failure, and death. So far, there are more than 30 different proteins, which have been identified as amyloidogenic—out of which at least 17 can cause systemic disease (1).

The most common forms of amyloidosis infiltrating the human heart (cardiac amyloidosis, CA) are (i) immunoglobulin light chain (AL) (2) and (ii) transthyretin amyloidosis (ATTR) (3). As a different type, secondary or amyloid A (AA) amyloidosis is caused by overproduction and accumulation of the acute-phase protein “serum amyloid A” (SAA) that can be highly expressed in patients with chronic inflammation, cancers or (auto)inflammatory diseases (4). The incidence of AA amyloidosis, in particular in developed countries, is low since this disorder only occurs as a long-term complication of rather severe chronic inflammatory disorders that in turn are mostly well-managed in developed countries—in case of timely detection.

To identify the underlying inflammatory process in newly diagnosed AA amyloidosis, several factors ranging from genetic variation to ancestral history of patients have to be taken into consideration. For example, SAA1 genotype is one of those predisposing factors supposed to affect amyloidogenesis (e.g., SAA1.1 in Caucasians and SAA1.3 in Japanese homozygotes) (5). Likewise, if amyloidosis is suspected, even in asymptomatic members of certain origins (for e.g., Turkish, Armenian, Greek, Spanish) familial Mediterranean fever (FMF) should be ruled out as a possible underlying cause of AA amyloidosis (6).

Available knowledge indicates that SAA does not affect the human heart directly, and significant clinical cardiac manifestations are rare. However, it has been shown that SAA may be deposited in cardiac tissue (7). If present, it can lead to severe ventricular wall thickening and subsequent stiffness resulting in a restrictive pattern as well as ventricular arrhythmias as major cardiac manifestations (8). Although CA, hypertrophic cardiomyopathy (HCM) and hypertensive heart disease (HHD) can be suspected as underlying disease in case of left ventricular (LV) hypertrophy of unknown origin based on patient history, symptoms, ECG and echocardiography, only myocardial tissue characterization by multi-parametric CMR (comprising late-gadolinium-enhancement imaging and novel mapping-based approaches) allows to safely differentiate between different forms of LV hypertrophy. Recently, Chatzantonis et al. showed a high diagnostic yield of CMR for the non-invasive diagnosis of cardiac ATTR amyloidosis based on the characteristic pattern of LGE in those patients (9). Surprisingly, even an extensive literature search for non-invasive imaging approaches to diagnose cardiac AA amyloidosis did not result in any landmark studies or even case reports illustrating the potential use of CMR imaging.

In the present report, the use of multi-parametric CMR imaging was crucial to diagnose cardiac involvement in case of systemic AA amyloidosis. We present two similar cases of AA amyloidosis in whom cardiac involvement was suspected based on CMR findings and subsequently proven by biopsy work-up. Importantly, both cases showed only a very subtle pattern of LGE—not resembling the well-known characteristic pattern of LGE that can be found in those patients with ATTR and AL amyloidosis. However, additional ECV measurement based on T1-mapping was highly suggestive of an ongoing infiltrative process in the extracellular space of the myocardium.

## Methods and Materials

Early diagnosis of cardiac involvement in amyloidosis can be challenging due to unspecific or missing clinical manifestations. Although non-invasive imaging modalities such as echocardiography (with speckle-based strain measurement), bone scintigraphy (10) as well as multi-parametric CMR are widely used today and promise both early and specific detection of cardiac amyloidosis (11, 12), invasive EMB still constitutes the gold-standard for the final diagnosis of non-ischemic heart diseases including cardiac amyloidosis (13). Noteworthy, imaging modalities such as bone scintigraphy and multi-parametric CMR were shown to be valuable tools in differentiating cardiac ATTR from AL amyloidosis if additional information on monoclonal protein studies are available and promise early detection of cardiac amyloidosis (9, 14). However, no convincing non-invasive imaging findings have been described so far regarding the detection of cardiac involvement in case of AA amyloidosis.

CMR has been established as a robust diagnostic tool for the work-up of different cardiac diseases including LV hypertrophy of unknown origin and was recently shown to offer a very high diagnostic yield regarding the diagnosis of cardiac ATTR and AL amyloidosis (15, 16). An overview on established diagnostic techniques and staging concepts for patients with CA was summarized recently (17). However, imaging parameters for the detection of cardiac involvement in case of AA amyloidosis are still not established. In the present report, we propose some CMR-based techniques to detect/suspect cardiac AA amyloidosis. The details of patient characteristics, cardiac imaging as well as EMB work-up are explained in the section separately.

### Patient Characteristics

Both subjects were Caucasian males who presented with end stage renal disease (ESRD) requiring dialysis. The first patient was a 44-year-old young male patient with terminal renal insufficiency and was on hemodialysis since 08/2018. A rectal biopsy performed in 11/2019 had revealed SAA depositions. Echocardiography showed a moderate to severe LV hypertrophy with preserved ejection fraction. Additional EMB samples were taken in this patient and confirmed the presence of cardiac AA amyloidosis. Subsequent genetic testing revealed heterozygosity for 2 pathogenic variants of the MEFV-gene and supported the diagnosis of FMF as a plausible cause of AA amyloidosis.

The second subject, a 53-year-old male and tobacco-smoking patient, was initially referred from a regional hospital to our university hospital following clinical deterioration of the general condition during dialysis in addition to new-onset of diffuse abdominal pain. Incidental findings comprised a pericardial effusion with a maximal diameter of 2 cm. Noteworthy, he was treated with colchicine since his childhood due to suspected MFM. This treatment was stopped in 2012 due to onset of terminal renal insufficiency leading to haemodialysis. The suspected diagnosis of FMF was confirmed by molecular genetic analysis as an underlying inflammatory cause after SAA deposits were detected in EMB samples. In addition, liver biopsies were also performed and confirmed the aforementioned results. Details of the respective patient characteristics can be found in Table 1. Written informed consent was obtained from all the patients for the publication of any potentially identifiable images or data included in this article and the study protocol conforms to the ethical guidelines of the 1975 Declaration of Helsinki.

**Table 1:** Patient characteristics.

**Parameter**	**Patient 1**	**Patient 2**
Age (years)	44	53
Sex	Male	Male
BMI (kg/m^2^)	21.6	21.1
Inflammatory extracardiac diseases	MFM, recurrent fevers, pleurisy, spontaneous bacterial peritonitis, Hepatitis B	MFM, mild recurrent fevers, abdominal and joint discomforts
Current medication	Ca^++^ channel blocker, beta-blocker, colchicine, dihydralazine, ACE-inhibitor, aspirin, atorvastatin, pregabalin, pramiprexol	Ca^++^ channel blocker, beta-blocker, colchicine, iron substitution, ACE-inhibitor
Heart rate	56	61
Blood pressure (mmHg), sys/dia	145/73	110/65
Temperature (°C)	36.8	36.4
C-reactive protein (mg/dl) [<0,5]	2.2	8
Hematocrit (Pre-Dialysis)	26	31
Leukocyte count [3.9–10.9 × 10^9^/L]	5.54	18.84
CK (U/L) [<174]	143	574
CK-MB (U/L) [<24]	13	172
eGFR (CKD-EPI) [> 90]	7	8
NT-proBNP (pg/ml) [<172]	> 35.000	5.740
**Major CMR findings**		
LV-EF (%)	56	66
LV-EDVi (ml/m^2^)	111	102
LV-ESVi (ml/m^2^)	49	35
LV mass (g/m^2^)	98	87
Max. thickness of the septal wall (mm)	16	13
RV-EF (%)	56	67
Global native T1 [950–1,050 m]	1.098	1.062
Basal septal native T1 [950–1,050 m]	1.108	1.082
Basal lateral native T1 [950–1,050 m]	1.067	1.112
Global native T2 [50–56 m]	58	56
Basal septal T2 [50–56 m]	56	58
Basal lateral T2 [50–56 m]	54	53
Global ECV [25–31%]	36	44
Septal ECV [25–31%]	39	42
Lateral ECV [25–31%]	36	48

*All data are from the day (± 3) of CMR examination, if not mentioned otherwise. Units are mentioned in small brackets (). Normal range of values are mentioned in large brackets []. BMI, body mass index; FMF, familial mediterranean fever; CMR, cardiovascular magnetic resonance; CK, creatine kinase; CK-MB, creatine kinase myocardial band; eGFR (CKD-EPI), estimated glomerular filtration rate according to chronic kidney disease epidemiology collaboration; NT-proBNP, N-terminal pro brain natriuretic peptides; ECV, extracellular volume fraction*.

### Endomyocardial Biopsy Protocol

EMB specimens were obtained from the LV free wall (*via* a femoral artery access site using a 7F long-sheath without angulation following a retrograde approach) as described previously (9). Continuous ECG, blood pressure and pulse oximetry monitoring were performed throughout the whole procedure. Fluoroscopy was used for guidance of the long-sheath and bioptome, and for targeting the region of interest—which was defined in advance by non-invasive CMR. Six EMB samples were collected from the LV. Post-procedural echocardiography was performed to rule out or detect a pericardial effusion possibly caused by the biopsy procedure.

### Histological and Immunohistochemical Analyses

EMB samples were fixed in 4% buffered formaldehyde for histology and immunohistochemical staining, or in RNAlater (Ambion Inc., Foster City, USA). EMBs were considered positive for viral infection if viral genomes were detected by nested PCR/reverse transcription PCR, as published previously (18). EMB specimens were stained with Congo red for the diagnosis of cardiac amyloidosis and examined by light microscopy. In addition, Congo red staining was demonstrated by typical green birefringence under cross-polarized light as described earlier (19).

### CMR Acquisition, T1/T2 and ECV Measurements

CMR studies were performed on a 1.5-T system (Ambition, Philips Healthcare, Best, The Netherlands). CMR data acquisition was performed according to the standardized protocol suggested by the Society for Cardiovascular Magnetic Resonance (SCMR) (20). Our CMR protocol comprised a cine steady-state free precession pulse sequence for ventricular function and a two-dimensional (2D) inversion recovery fast spoiled gradient-echo sequence 10 to 15 min after administration of a gadolinium-based contrast agent (Gadobutrol 0.10 mmol/kg) for detection of myocardial pathology. Moreover, a modified Look-Locker inversion recovery (MOLLI) T1-mapping sequence was applied in basal, mid and apical short-axes prior to contrast agent administration and ~20 min thereafter to determine native T1 and ECV values as described previously (21). Motion corrected native and post-contrast T1 maps were generated from the pre- and post-contrast T1 sequences. In each short-axis T1 map the endo- and epicardial contours were manually drawn. For T2-mapping, similar to T1, motion corrected but only native pre-contrast sequences were used. Additionally, for ECV calculation, a region of interest was drawn in the blood pool (avoiding the papillary muscles) in all analyzed T1 maps. Motion corrected and segmented ECV maps were generated from the native and post-contrast segmented T1 maps, using the patient's hematocrit level as described by us elsewhere (21). “Global” T1, T2 and ECV values were calculated by averaging all 16 segments from three short-axis slices.

## Results

### CMR Findings

**Figure 1:**
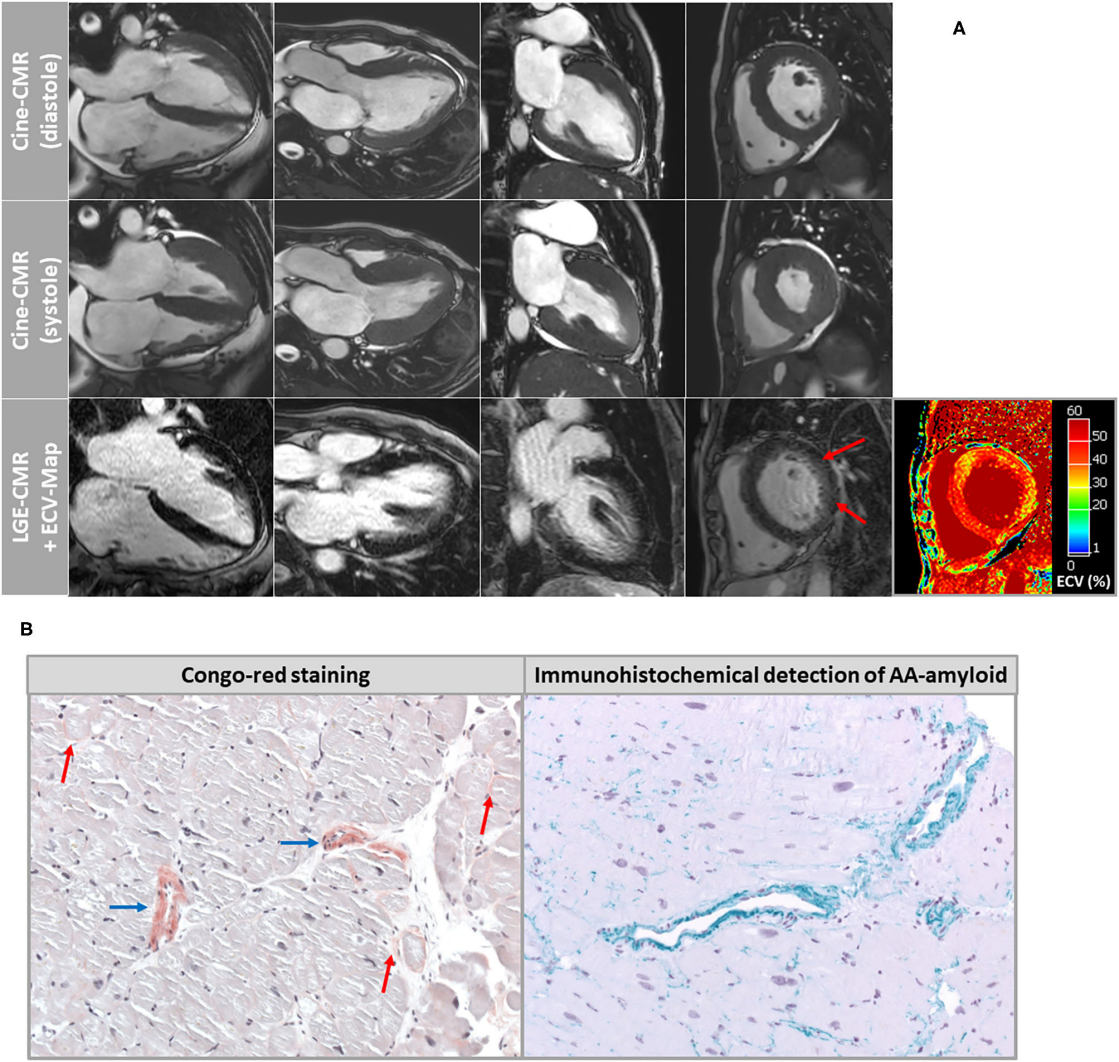
**(A)** Cardiovascular magnetic resonance (CMR) images of patient-1 showing cine images in diastole and systole as well as corresponding late-gadolinium-enhancement (LGE) and extracellular volume fraction (ECV) maps of the myocardium. A very subtle and rather diffuse pattern of LGE was detected in the basal segments of the LV lateral wall. However, ECV maps illustrated highly elevated myocardial ECV values in the septal and lateral wall segments. **(B)** Histopathological images of endomyocardial biopsy (EMB) samples that were taken in patient-1 from the left ventricular free wall. Congo-red staining showed a diffuse pattern of interstitial amyloidosis (red arrows) in addition to marked accumulation of amyloid deposits within the vessel walls (blue arrows). The subtype AA amyloidosis was proven by specific immunohistochemistry.

**Figure 2:**
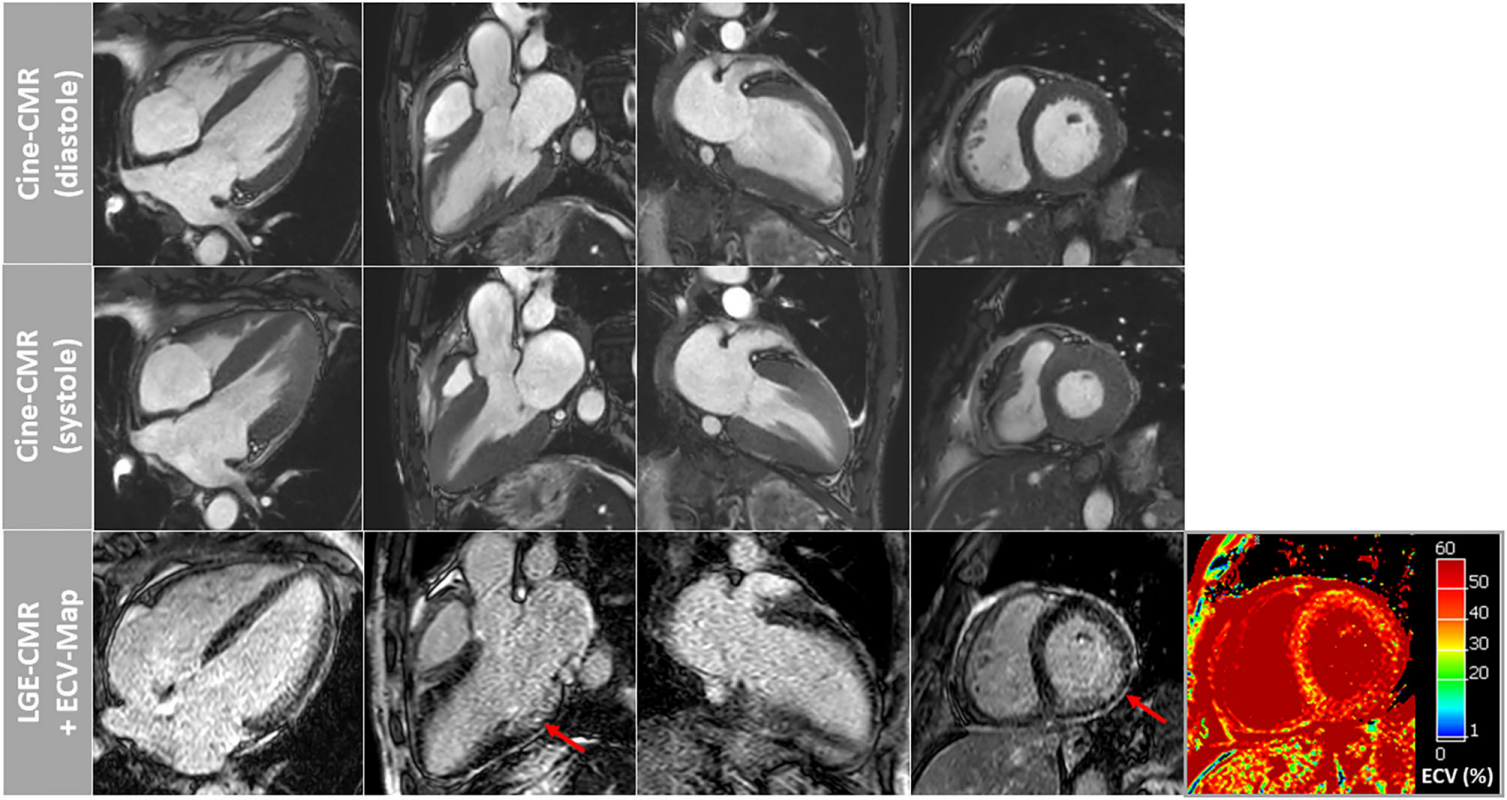
Cardiovascular magnetic resonance (CMR) images of patient-2 showing cine images in diastole and systole as well as corresponding late-gadolinium-enhancement (LGE) and extracellular volume fraction (ECV) maps of the myocardium. A slightly more pronounced non-ischemic pattern of LGE was observed in this patient in the basal to midventricular inferior/inferolateral segments of the LV wall (red arrows). Again, ECV maps illustrated highly elevated myocardial ECV values in the septal and lateral wall segments.

Both patients showed similar results regarding cine-imaging: LVs were slightly dilated, mild to moderately hypertrophied with preserved systolic function. Right ventricles (RV) showed no abnormalities with regard to size and function. Regarding myocardial tissue characterization, a subtle and rather diffuse pattern of LGE was detected in the basal segments of the LV lateral wall. Moreover, a little pericardial effusion was observed (Figure 1A). Patient 2 showed a slightly more pronounced non-ischemic pattern of LGE in the basal to midventricular inferior/inferolateral segments of the LV wall (Figure 2). In addition, some pericardial enhancement suggestive of pericarditis as well as pleural effusion suggestive of poly-serositis, most likely in the setting of the underlying disease, were present. Importantly, myocardial mapping measurements resulted in a marked elevation of ECV-values as well as slight elevation of native T1-values predominantly in the basal septal and lateral LV walls, and rather normal T2-values in both patients—indicating a rather chronic, non-inflammatory and most likely infiltrative process (Figures 1A, 2). Detailed mapping results are illustrated in Table 1.

### Histological and Immunohistochemical Findings

Based on histopathology, there was no evidence of active lymphocytic myocarditis. Using Congo-red staining and immunohistochemistry, the presence of cardiac AA amyloidosis was confirmed with a very subtle and diffuse amyloid pattern in the extracellular space and a marked vascular involvement (Figure 1B). In addition, other possible diagnoses such as hypertensive heart disease, hematochromatosis, classic HCM, glycogenosis, Fabry disease or dilated cardiomyopathy were ruled out. Amyloid subtyping was achieved with immunohistochemical staining and showed a pronounced positive reaction for amyloid A (Figure 1B). The amyloid deposits were negative regarding lambda and kappa light chain antibodies. Moreover, there was no immunohistological evidence for the presence of ATTR amyloidosis.

## Discussion

Cardiac involvement in case of AA amyloidosis is not common and rather challenging to diagnose. An extensive literature search regarding the diagnostic value of non-invasive imaging modalities to diagnose cardiac AA amyloidosis did not result in any landmark studies nor in convincing case reports. Obviously, echocardiography is a widely available tool that has improved significantly the diagnosis of CA after the introduction of speckle-tracking-based strain analysis, however, is still lacking high sensitivity as well as specificity (21–23). In contrast, bone scintigraphy and targeted PET studies offer higher sensitivity and specificity regarding the detection of CA, and even allow to differentiate between AL and ATTR amyloidosis if additional monoclonal protein studies are available. However, convincing reports showing detection of AA amyloidosis by bone scintigraphy or PET are still missing.

Another modality, non-invasive multi-parametric CMR imaging, has gained wide acceptance within the last years and allows (amongst others) sophisticated work-up of non-ischemic cardiomyopathies, and in this context to differentiate between CA and HCM based on the pattern of LGE. While the LGE pattern in case of CA is rather diffuse, starts mostly from the subendocardial layer of the basal segments and eventually spreads to all myocardial layers and segments (16, 24), classic HCM is characterized by a patchy and more focally accentuated LGE pattern predominantly occurring in the most hypertrophic septal segments of the left ventricular myocardium (25, 26). Additionally, pre- and post-contrast T1-mapping applied for tissue characterization by measurement of the intrinsic T1-relaxation time of the myocardium allows to determine the ECV value of the myocardium (27). Similarly, T2-mapping is a promising technique for both visualization and *in vivo* quantification of myocardial edema suggestive of myocardial inflammation (28). Myocardial edema is characterized by an increase in myocardial water content (per voxel myocardium) and thereby causes longer T2-relaxation times (29).

Several previous studies have shown that measurement of native T1 and ECV values are useful tools for the work-up of hypertrophic cardiac phenotypes of unknown origin (30). The degree of increase in global native T1 and ECV values is consistently lower in HCM as compared to CA as there is an extensive, diffuse amyloid infiltration of the extracellular space in case of CA (31) while HCM mostly shows focally accentuated interstitial fibrosis in hypertrophied septal segments (32). Noteworthy, our present report clearly illustrates that only a very subtle pattern of LGE—not resembling the well-known characteristic pattern of LGE that can be found in those patients with ATTR and AL amyloidosis (!)—is seen in case of AA amyloidosis. However, additional ECV measurements based on pre- and post-contrast T1-mapping resulted in surprisingly high myocardial ECV values—suggesting an ongoing infiltrative process in the extracellular space of the myocardium.

Obviously, both patients were on hemodialysis and in principle, “uremic” cardiomyopathy may manifest with LV hypertrophy as well as diffuse (interstitial) myocardial fibrosis that in turn may lead to increased myocardial native T1 and ECV values. In this context, Hayer et al. recently studied *N* = 134 non-diabetic patients with chronic kidney disease (CKD) stages 2 to 5 without myocardial ischemia at 1.5-Tesla and found native T1 values of 966 ± 21 ms in stage 2 patients vs. 994 ± 33 ms in stage five patients (*p* < 0.001) (33). In addition, Hayer et al. studied *N* = 24 patients with end-stage renal disease (ESRD)—again at 1.5-Tesla—and documented septal native T1-values of 1002 ± 30 ms and a myocardial ECV value of 28 ± 2% (34). Unfortunately, comprehensive data regarding myocardial ECV are limited in ESRD patients since gadolinium-based contrast agents are rarely used in those patients. Of course, we cannot exclude an “additional” effect of ESRD on LV hypertrophy, native T1 and ECV values in our patients. However, we believe that such an “additional” or “confounding” effect—apart from cardiac manifestation of AA amyloidosis—was limited due to the following simple reason: The native T1- and ECV-values that were measured in the myocardium of our patients (at 1.5-Tesla) were substantially higher (!) compared to the aforementioned reference values in ESRD patients published recently.

In summary, unlike other well-known subtypes of amyloidosis (such as ATTR and AL), a characteristic pattern of diffuse LGE is not observed in case of AA amyloidosis based on CMR. However, measurement of myocardial ECV—in addition to myocardial T1-/T2-mapping—may be of paramount diagnostic value to detect cardiac involvement in case of AA amyloidosis—despite a missing characteristic LGE pattern. Therefore, we recommend a comprehensive multi-parametric CMR approach comprising the combined analysis of T1-/T2-mapping, measurement of ECV and careful assessment of LGE-images in patients with AA amyloidosis and suspected cardiac involvement.

## Data Availability Statement

The original contributions presented in the study are included in the article/supplementary material, further inquiries can be directed to the corresponding author.

## Ethics Statement

Ethical review and approval was not required for the study on human participants in accordance with the local legislation and institutional requirements. Written informed consent for participation was not required for this study in accordance with the national legislation and the institutional requirements. Written informed consent was obtained from the individual(s) for the publication of any potentially identifiable images or data included in this article.

## Author Contributions

BC devised the project and the main conceptual idea, and wrote the manuscript. SD contributed to sample and data collection. MB and CM provided critical feedback and helped shape the research and analysis. KK performed the histological and immunohistochemical analyses. AY supervised the work, contributed to the interpretation of results, provided critical feedback and helped shape the research, analysis, and manuscript. All authors contributed to the article and approved the submitted version.

## Conflict of Interest

The authors declare that the research was conducted in the absence of any commercial or financial relationships that could be construed as a potential conflict of interest.

## Publisher's Note

All claims expressed in this article are solely those of the authors and do not necessarily represent those of their affiliated organizations, or those of the publisher, the editors and the reviewers. Any product that may be evaluated in this article, or claim that may be made by its manufacturer, is not guaranteed or endorsed by the publisher.
